# Deteriorated biomechanical properties of human hypertrophied septum in response to cardiomyocyte enlargement, overexpressed collagen, and disarrayed microstructures

**DOI:** 10.3389/fbioe.2025.1620594

**Published:** 2025-06-02

**Authors:** Katherine M. Copeland, Houjia Chen, Uday Chintapula, Milad Almasian, Duc Khang Chung, Alan M. Taylor, Yichen Ding, Gaurav Sharma, Michael E. Jessen, Yi Hong, Kytai T. Nguyen, Matthias Peltz, Pietro Bajona, Jun Liao

**Affiliations:** ^1^ Department of Bioengineering, University of Texas at Arlington, Arlington, TX, United States; ^2^ Department of Bioengineering, University of Texas at Dallas, Richardson, TX, United States; ^3^ Department of Cardiovascular and Thoracic Surgery, University of Texas Southwestern Medical Center, Dallas, TX, United States

**Keywords:** emerging tools, concepts, and applications in multi-scale mechanobiology, hypertrophic cardiomyopathy, human hypertrophied septum, biomechanical deterioration, microstructural abnormalities, collagen overexpression

## Abstract

Hypertrophic cardiomyopathy (HCM) is often caused by genetic mutations, resulting in abnormal thickening of ventricular muscle, particularly the septum, and causing left ventricular outflow tract (LVOT) obstruction and inferior cardiac performance. The cell and microstructural abnormalities are believed to be the cause of the altered tissue mechanical properties and inferior performance. However, there is a lack of detailed biomechanical assessments of human hypertrophied septum and a lack of understanding of the structural-mechanical relationship between altered biomechanical properties and cellular hypertrophy, fibrotic overexpression, and microstructural disruptions. In this study, we performed thorough biomechanical and microstructural characterizations on the human hypertrophied septum and compared this with healthy septum. We found that the hypertrophied human septum was stiffer at the initial phase of tissue loading, but less nonlinear, less stiff in the linear region, and much weaker in mechanical strength when compared to the healthy human septum. The fibrosis-induced initial stiffening in the hypertrophied septum paradoxically coexists with compromised mechanical strength and integrity under physiological demands, correlating with the clinical observations of diastolic dysfunction and susceptibility to myocardial damage in HCM patients despite ventricular wall thickening. We also discovered that the human hypertrophied septum had significantly larger stress relaxation and slightly larger creep when compared to healthy septum. Moreover, the abnormal, disorganized cell-collagen microstructures in the hypertrophied septum make short-term stress release more difficult and require longer relaxation times to reach equilibrium. Biaxial testing performed at the initial phase of tissue loading showed that both the healthy septum and hypertrophied septum had nonlinear anisotropic stress-strain behavior and confirmed that, in the longitudinal direction, the hypertrophied septum was stiffer than the healthy septum. Our microstructural quantifications via histology and light-sheet microscopy revealed that (i) the heterogeneous cardiomyocyte enlargement and disarray, combined with disorganized collagen overexpression, create a mechanically inefficient tissue architecture in the hypertrophied septum, and (ii) the observed cell-collagen microstructural disruptions provide mechanistic explanations for the deteriorated biomechanical properties. Our viscoelastic mechanical data and microstructural characterizations build a strong foundation to understand the altered tissue behavior of the hypertrophied septum, the degree of deviation from the normal septum, and the underlying structural mechanisms.

## 1 Introduction

Hypertrophic cardiomyopathy (HCM) is a genetic disease caused by mutations in contractile sarcomeric proteins that result in abnormal thickening of the ventricular heart muscle, affecting particularly the septum that separates the left and right ventricles (Roberts and Marian, 2001; Kimura et al., 1997; Semsarian et al., 2015; Kraft et al., 2013; Maron et al., 2012). The most common mutations of HCM are found in *MYBPC3* (myosin binding protein C3) and *MYH7* (β-myosin heavy chain) genes (Maron et al., 2012; Maron, 2002; Maron et al., 1987; Spirito et al., 1997; Marian and Braunwald, 2017). These mutations account for ∼50% of all cases of HCM. HCM can affect as many as 1/200 people, making it the most common genetic cardiomyopathy in the general population (Roberts and Marian, 2001; Marian and Braunwald, 2017; Elliott et al., 2000; Bagnall et al., 2018). Approximately 70% of HCM patients experience hypertrophic obstructive cardiomyopathy with clinical manifestations, such as larger than 13 mm left ventricular wall thickness, left ventricular outflow tract obstruction (LVOTO), normal or increased ejection fraction, atrial or ventricular arrhythmias, heart failure, and/or sudden cardiac death (SCD) (Roberts and Marian, 2001; Semsarian et al., 2015; Maron, 2002; Marian and Braunwald, 2017; Okutucu et al., 2016; Helms et al., 2014; O'Mahony et al., 2014; Roberts et al., 1992; Klues et al., 1992).

Pharmaceutical drugs, such as beta blockers and calcium channel blockers, are used to alleviate symptoms (Spirito et al., 1997; Kosutic, 2005; Rosso et al., 2007; Rosing et al., 1985; Serfas D and Lorell, 1983; Rosing et al., 1979). More recently, myosin inhibitors such as mavacamten have achieved regression of hypertrophied muscle (Braunwald et al., 2023; O'Malley, 2024). When medications alone are not sufficient to relieve symptoms, the current gold standard, septal myectomy, is performed to surgically remove excess muscle in an open heart surgery (Ommen et al., 2005; Smedira et al., 2008; Maron et al., 2017). The removal of bulged, overgrown septum relieves obstruction of blood flow and improves the function of the left ventricle outflow tract. For inoperable patients, cardiologists employ a minimally invasive catheterization technique known as alcohol septal ablation (ASA), in which 1–4 mL of pure alcohol is injected via a septal perforator (artery) to destroy a part of the overgrown septal muscle (Alam M and Lakkis, 2006; Talreja DR et al., 2004; Nagueh et al., 2011; Holmes and Nishimura, 2005; Veselka et al., 2010; ten Cate et al., 2010). However, both septal myectomy and ASA have complications such as infection, bleeding, cardiac tamponade, arrhythmia, ventricular fibrillation, and heart block.

Hypertrophic cardiomyopathy presents considerable clinical challenges due to its abnormal anatomy, disruption of the cardiac mechanics, progressive disease, and limited treatment options. Microstructurally, hypertrophic cardiomyopathy is characterized by the significantly enlarged size of hypertrophic cardiomyocytes, the disorganized arrangement of these cells, and overexpressed collagen that forms a fibrotic microenvironment (Guttmann et al., 2014; O'Hanlon R et al., 2010). Both cellular hypertrophy and fibrosis contribute to the thickening of the ventricular wall and the overgrowth of the septum, which significantly alter the macroscopic anatomy of the heart, myocardial contractility, as well as the ventricular mechanics during diastolic relaxation and systolic contraction.

For instance, it has been shown that the restricted outflown of blood due to LVOTO increases the mechanical stress experience by the heart (Roberts et al., 1992; Klues et al., 1992). The elevated mechanical stress is also believed to be one of the major contributing factors in the development of a fibrotic collagenous microenvironment in HCM septum and the ventricular wall (Varnava et al., 2000; Maron et al., 2003). One of the reported biomechanical abnormalities is the circumferential myocardial shortening of ventricular muscle during contraction, which is directly involved in the preservation of left ventricular systolic performance in HCM (Urbano-Moral et al., 2014). Moreover, the reduced left ventricular strain, i.e., the decreased myocardial deformation due to the thickening of the wall, has also been associated with poor cardiac outcomes, which consequently results in heart failure and mortality (Hinojar et al., 2017).

HCM is a complex condition influenced by abnormalities in genetics, cell biology, fibrosis, and tissue mechanics, and these factors and are often intertwined. There are still many unknowns about HCM disease, and one of the critical knowledge gaps is the incomplete understanding of the structural and mechanical relationship of human hypertrophic cardiac tissue. In this study, we focused on human hypertrophied septum to examine how the cardiomyocyte hypertrophy and the altered extracellular matrix (ECM) microenvironment affect the biomechanical properties, compared to healthy human septum.

## 2 Materials and methods

### 2.1 Acquisition of hypertrophied septal tissues and healthy septal tissues

Hypertrophied septal samples were obtained from the septal myectomy of 14 patients who underwent surgery at the University of Texas Southwestern Medical Center (IRB STU 082017-072). Patients include both male and female and range in age from 19 to 71 years. Most patients presented with mild to moderate mitral valve regurgitation and septal thicknesses ranging from 17 mm to 32 mm. After therapeutic myectomy was completed, a portion of the resected pathologic specimen was immediately placed in a container filled with cardioplegia solution and transferred in an iced cooler to UTA for histology and biomechanical characterizations, while the remainder of the pathologic specimen was sent to pathology for routine review. Upon arrival, samples were examined to determine orientation and dissected for various testing purposes based on its tissue size. In this study, the longitudinal direction (LD) is determined as the axis that follows along the length of the trabeculae carneae and the cross-sectional direction (CD) is defined as the axis transverse to the trabeculae. Healthy human septal tissues were obtained from hearts recovered at the local organ procurement organization (Southwest Transplant Alliance, Dallas, TX) as part of an approved research protocol with matched age range. These hearts were deemed unsuitable for transplantation but had normal cardiac function and chamber size on echocardiography.

Septal tissues were then dissected for uniaxial tension testing and biaxial testing. Samples prepared for uniaxial testing had dimensions of ∼15 mm (LD) × 10 mm (CD) × 3 mm (t), with the longest dimension aligned with the LD. All samples were further trimmed to achieve a dog-bone shape and 5 mm width. For biaxial mechanical testing, samples were dissected into square shapes, with dimensions of 10 mm (LD) × 10 mm (CD) × 3 mm (t). The sample dimensions were determined based on myectomy tissue size constraints, need of tissue allocation for various tests, and our protocol routine. The remaining parts of the non-tested samples were used for histological evaluation. All biomechanical testing was performed in a 1X Phosphate Buffered Saline (PBS) bath.

### 2.2 Uniaxial tensile testing and characterizations of tissue viscoelasticity

Besides stress-strain behavior up to tissue failure, stress relaxation and creep are two important viscoelastic properties of soft tissues. Stress relaxation describes the time-dependent gradual stress decay in tissues when a constant strain is applied and maintained (Abdallah et al., 2019). Creep describes defined the time-dependent progressive deformation of tissues when the sample is subjected to a constant load (Coffin and Fellers, 2001). In the experiment, longitudinally oriented tissue samples were mounted on the TestResources Universal Testing Machine (Shakopee, MN). For uniaxial tensile failure testing (n = 7), Tissue samples were subjected to 10 cycles of 10% preconditioning (Ahmad et al., 2018; Humphrey et al., 1990; Novak et al., 1994; Humphrey and Yin, 1987; Valdez‐Jasso et al., 2012), and then loaded until failure (tissue breakage). For stress relaxation and creep tests (n = 5), the following testing protocols were conducted: (1) Stress relaxation: After preconditioning, HCM and healthy samples were loaded to 100 g, and the achieved strain was kept constant while recording the stress decay up to 15 min (900 s). (2) Creep: HCM and healthy samples were loaded to 100 g and kept at this constant load while deformation was continuously monitored up to 15 min. The sample ramping rate for all the tests was 0.5 mm/s. Engineering stress was calculated by normalizing the applied force to the initial cross-sectional area of the tissue sample. Engineering strain was calculated by normalizing the amount of deformation to the initial dimension.

### 2.3 Biaxial mechanical testing

A custom-built biaxial mechanical testing system was used to characterize the anisotropic mechanical behavior of HCM septum and healthy septum under physiologically relevant loading conditions (Ahmad et al., 2018; Brazile et al., 2021; Shi et al., 2019; Wang et al., 2013; Zhang et al., 2010). Following dissection, square samples (HCM: n = 6; Healthy: n = 3) were mounted on the biaxial testing machine with one edge aligned with LD direction and the other edge aligned with CD direction. Each side of the square sample was secured with four stainless steel hooks that were attached to two loops of 000 polyester sutures. Four fiducial markers were glued to the center of the square sample with minimal cyanoacrylate adhesive to record the real time tissue deformation via a CCD camera. Loads along two directions were measured by two load cells mounted orthogonally. The tissue sample was preconditioned for 10 cycles by applying biaxial tension up to 30 N/m along both LD and CD directions. After preconditioning, the equibiaxial tension protocol of T_LD_:T_CD_ = 30:30 N/m was performed to capture the biaxial behavior. The septal tissue samples were loaded only up to 30:30 N/m to avoid possible tissue tears at the hook sites at higher biaxial tension level. The biaxial tests thus captured the tissue behavior under physiologically relevant loading condition and at a relatively lower stress level (∼10 kPa).

### 2.4 Histology and microstructural analyses

Septal tissues for histological study were fixed in 10% neutral buffered formalin for 24 h and then processed through a standard histological preparation protocol, including alcohol-dehydration, xylene-clearance, and paraffin-embedment (n = 3). Embedded septal tissues were cut into 5 μm sections and subjected to Masson’s Trichrome staining, which stained heart muscle red and collagen blue (three slices used from each sample). Histological images were taken with a Nikon Eclipse Ti Series Microscope (Nikon) under bright light field. To analyze the size and orientation distribution of hypertrophied and healthy heart muscle cells, histological images were converted to 8-bit gray scale and subjected to thresholding to capture cell morphology using ImageJ (ImageJ, NIH, Bethesda, Maryland). The average cell size were quantified using ImageJ particle analyses, and cell orientation distribution were assessed using OrientationJ plugin in ImageJ (Rezakhaniha et al., 2012).

### 2.5 Light-sheet imaging

For light-sheet imaging, the iDISCO tissue clearing method employs a three-step process comprising dehydration, delipidation, and refractive index matching (Almasian et al., 2024; Ding et al., 2018). The tissue clearing method involves the complete removal of lipids and dehydration of the tissue, which largely minimizes light scattering. The organic solvent solution is then used to replace the dehydrated and delipidated tissue with refractive index matching to render heart transparent. The light-sheet was created with a cylindrical lens, and the electrically tunable lens (ETL) scanned the light-sheet focus which is synchronized with the active pixels on the scientific complementary metal-oxide-semiconductor (sCMOS) camera to generate a uniform light-sheet across the entire septum (Almasian et al., 2024; Ding et al., 2018). The septum was mounted in a holder attached to a four-axis motorized stage within a chamber filled with organic solvents. The stage continuously moved the sample across the detection axis at a constant velocity determined by the step size and the 2D image acquisition time, thereby enabling the acquisition of a 3D image stack of the heart. A 532 nm laser was used to illuminate the septum, and the autofluorescence emission was collected through a 0.25 NA objective lens with 4× magnification (pixel size of 1.625 µm) with a step size set to 2 µm. A customized LabVIEW control algorithm controlled and synchronized our in-house light-sheet microscope. (Sodimu et al., 2023). Volume rendering of the acquired images was done using the Amira software.

### 2.6 Statistical analyses

All values are reported as the mean ± standard deviation (SD), where a *p*-value less than 0.05 was considered as statistically significant. Unpaired two sample t-tests (equal variances, two-tailed) were performed to assess the statistical significance of potential differences between healthy and HCM septal tissues. All statistical analyses were conducted in RStudio (R Foundation for Statistical Computing, Austria).

## 3 Results

### 3.1 Tensile stress-strain behavior

**FIGURE 1:**
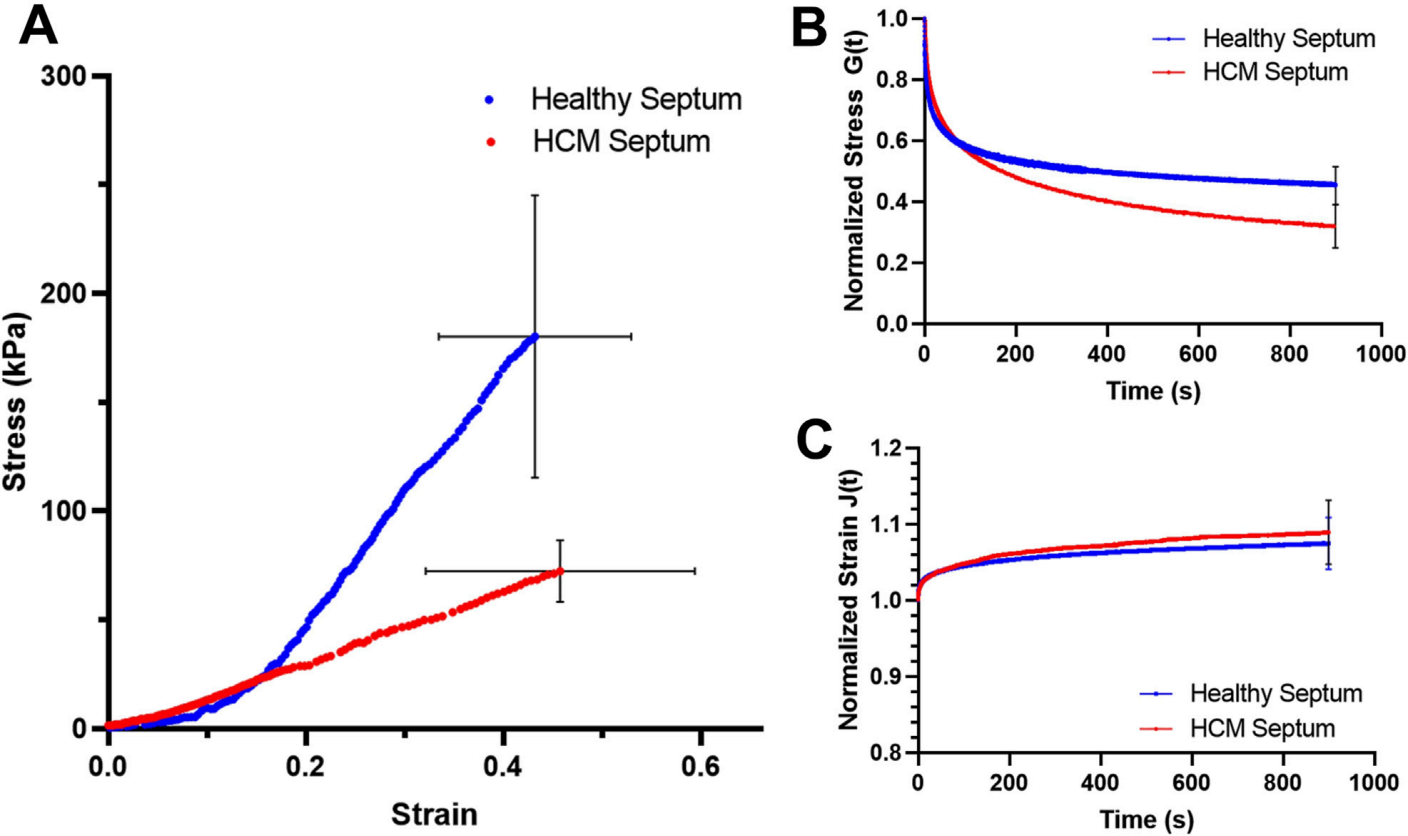
**(A)** Average stress-strain behavior of the healthy septum and HCM septum. **(B)** Average stress relaxation curves for the healthy septum and HCM septum; the final amount of stress decay at 15 min was 56.63% ± 6.29% for the healthy septum and 68.36% ± 9.85% for the HCM septum. **(C)** Average creep curves for healthy septum and HCM septum. The final amount of creep at 15 min reached 9.1% ± 1.0% for the healthy septum and 9.8% ± 4.2% for the HCM septum. Note that blue curves are for healthy septum and red curves are for HCM septum.

The averaged tensile stress-strain curves of the healthy septum showed a typical nonlinear concave upward stress-strain trend of most soft tissues (Figure 1A). The HCM septum, however, showed a stress-strain trend that was less nonlinear (Figure 1A). The HCM septum had an initial modulus that is higher than healthy septal tissues (healthy: 51.61 ± 19.93 kPa vs. HCM: 66.61 ± 38.48 kPa, *p = 0.35*), possibly due to the overexpressed fibrotic collagen associated with HCM. Interestingly, we found that the healthy septum had much higher mechanical strength than the HCM septum, demonstrated by the failure stress (healthy: 180.24 ± 64.88 kPa vs. HCM: 72.40 ± 14.17 kPa, p < 0.01). In the high strain region, the healthy septum was much stiffer than the HCM septum, as shown by the maximum tensile modulus of the tissues (healthy: 388.29 ± 188.61 kPa vs. HCM: 198.24 ± 106.68 kPa, p < 0.05). Overall, HCM septum was less nonlinear, stiffer at the initial phase of tissue loading, less stiff in the linear region, and much weaker in mechanical strength (Figure 1A; Table 1), indicating the deterioration of biomechanical properties of this disorganized, hypertrophic, and fibrotic heart muscle tissue.

**TABLE 1:** The comparison of the tensile mechanical parameters between healthy septum and HCM septum.

	Initial modulus (kPa)	Maximum modulus (kPa)	Failure stress (kPa)	Failure strain
Healthy Septum	51.61 ± 19.93	388.29 ± 188.61	180.24 ± 64.88	0.42 ± 0.10
HCM Septum	66.61 ± 38.48	198.24 ± 106.68	72.40 ± 14.17	0.46 ± 0.14
	*p = 0.35*	*p < 0.05*	*p < 0.01*	*p = 0.50*

### 3.2 Stress relaxation and creep

Healthy septum exhibited a stress decay of 56.63% ± 6.29% at 15 min, while the hypertrophied septum exhibited a much larger stress decay of 68.36% ± 9.85% at 15 min (*p* < 0.05) (Figure 1B). Both healthy septum and hypertrophied septum showed a small amount of creep over time. The final creep amount at 15 min reached 9.1% ± 1.0% for healthy septum and 9.8% ± 4.2% hypertrophied septum (Figure 1C).

We further assessed the stress relaxation rate and the creep rate in both the short-term and long-term periods. Note that the instant stress relaxation rate and creep rate were estimated from the average stress relaxation curve and the average creep curve, respectively. As a secondary variation, the time-dependent instant stress relaxation rate and creep only serve as a quantitative verification of the visual observation. Comparing data between HCM septum and healthy septum revealed subtle differences of viscoelastic behavior at various time points (Table 2).

**TABLE 2:** The instant change rates of normalized stress relaxation and the instant change rates of normalized creep estimated from the following four time regions: 0 s – 5 s, 40 s–50 s, and 200 s–250 s, and 800 s–900 s. The comparison between healthy septum and HCM septum gave an intuitive understanding how the HCM septum was different from the healthy septum in short-term viscoelastic response and long-term viscoelastic responses.

	Amount of normalized stress relaxation at 900 s (%)	Change rate of normalized stress relaxation (% per second)	at 0 s–5 s	at 40 s–50 s	at 200 s–250 s	at 800 s–900 s
Healthy Septum	56.63 ± 6.29		5.485	0.155	0.058	0.011
HCM Septum	68.36 ± 9.85		4.157	0.140	0.083	0.017

	Amount of normalized creep at 900 s (%)	Change rate of normalized creep (% per second)	at 0 s–5 s	at 40 s–50 s	at 200 s–250 s	at 800 s–900 s
Healthy Septum	9.1 ± 1.0		0.513	0.021	0.006	0.002
HCM Septum	9.8 ± 4.2		0.460	0.022	0.007	0.003

For stress relaxation, we noticed that (i) the healthy septum had a stress relaxation rate faster than the hypertrophied septum in the short-term relaxation period, which indicated the internal microstructures of the healthy septum were able to release their stresses more quickly and easily than the hypertrophied septum soon after the constant strain applied; (ii) the healthy septum, however, showed smaller stress relaxation rate in the long-term relaxation period when compared with the hypertrophied septum, indicating the internal microstructures of the healthy septum was able to stabilize early to approach equilibrium; on the other hand, the hypertrophied septum had internal microstructures that were likely more difficult to reach stabilization and hence continue stress decay at a higher rate than the healthy septum during this long-term relaxation period. Although not statistically significant, the hypertrophied septum showed a creep amount (9.8% ± 4.2%) that was slightly larger than the healthy septal tissues (9.1% ± 1.0%).

### 3.3 Biaxial mechanical behavior

**FIGURE 2:**
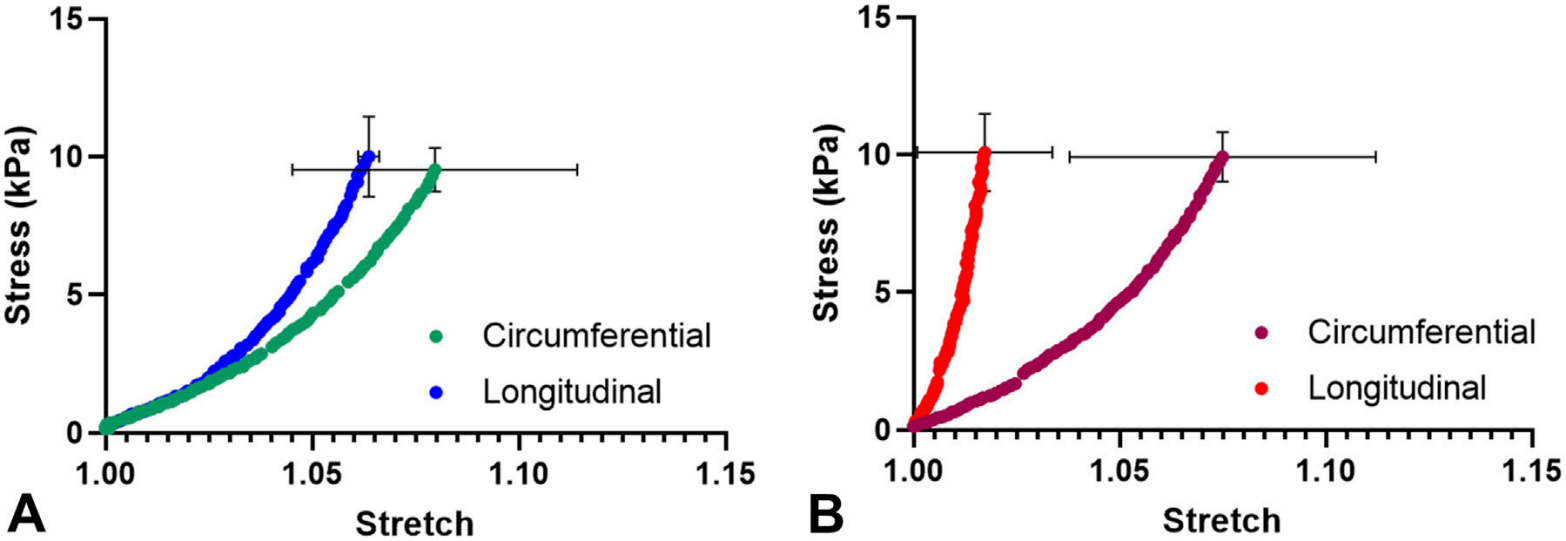
**(A)** Average biaxial mechanical behavior of the healthy septum. **(B)** Average biaxial mechanical behavior of the HCM septum.

Both the healthy septum and hypertrophied septum possessed nonlinear, anisotropic mechanical responses, with circumferential direction was less stiff than the longitudinal direction. Hypertrophied septum was found to be stiffer than the healthy septum in the longitudinal direction (Figure 2), which was consistent with the uniaxial stress-strain behavior observed in this tissue initial stress-strain region (Figure 1C). At 10 kPa stress level, the longitudinal extensibility of the hypertrophied septum was 1.7% ± 1.6%, while the longitudinal extensibility of the healthy septum was 6.4% ± 0.25% (*p < 0.05*). The circumferential direction appears similar between both groups, with extensibility at 7.5% ± 3.7% for the hypertrophied septum and 8.0% ± 3.4% for the healthy septum.

### 3.4 Quantification of microstructural abnormalities

**FIGURE 3:**
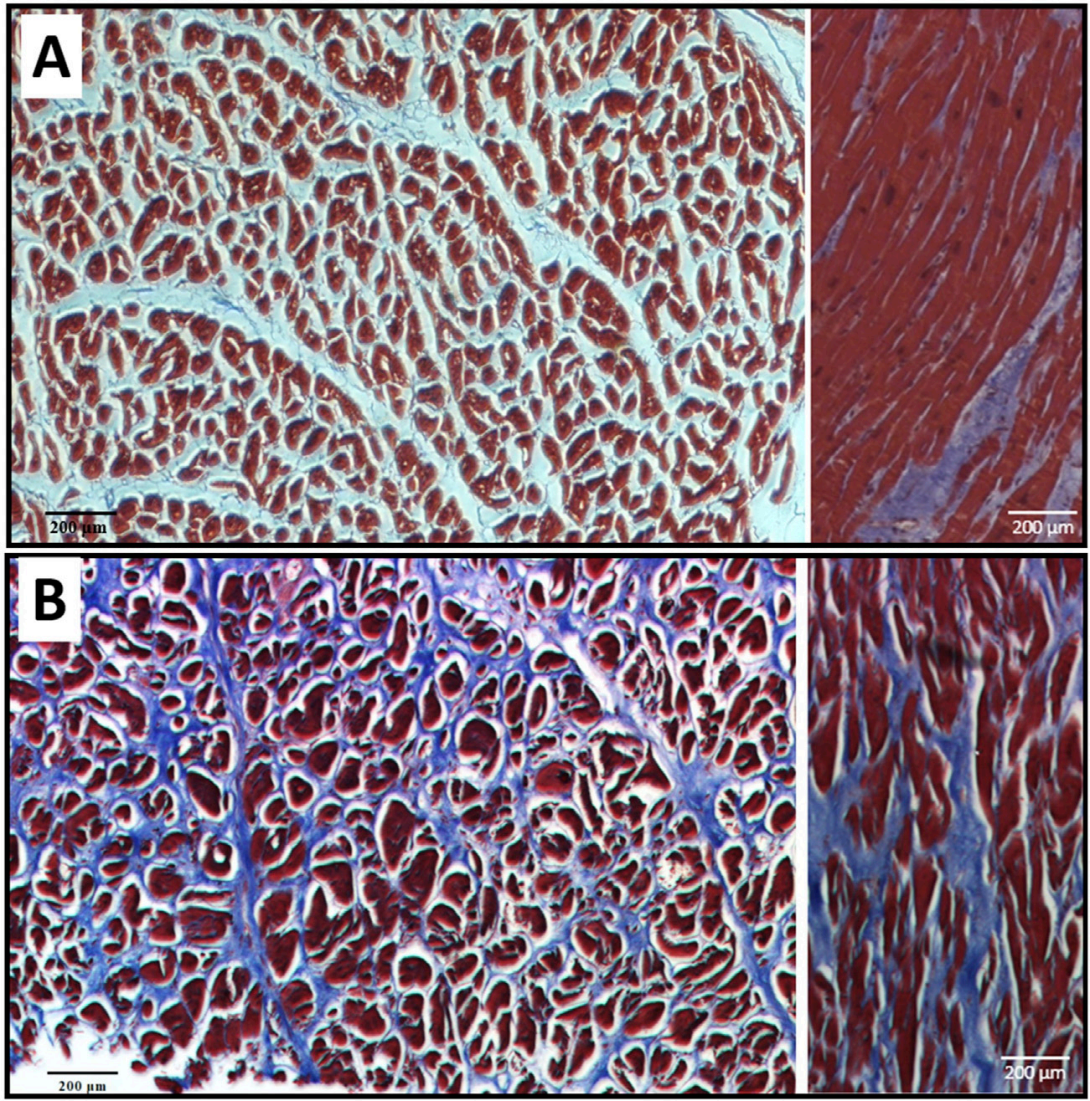
Masson’s trichrome staining revealed that the HCM septum had hypertrophic cardiomyocytes that were significantly larger and more disarrayed **(B)** when compared with the healthy cardiomyocytes **(A)**. The hypertrophic septum also had a higher amount of fibrotic collagen fibers **(B)**. For both panel **(A,B)**, the image on the left is a representative histology of a cross-sectional section, and the image on the right is a representative histology of a longitudinal section.

Histological images revealed that the hypertrophied septum had significantly enlarged and disarrayed cardiomyocytes, as well as overexpressed endomysial collagen and perimysial collagen when compared to the healthy septum (Figure 3). The cross-sectional view demonstrated that, in the healthy septum, cardiomyocytes have relatively uniform cell size and distribution (Figure 3A); on the contrary, most of the cardiomyocytes in the hypertrophied septum showed enlarged cell size and a non-uniform size distribution (Figure 3B). The disarrayed muscle fiber configuration in the hypertrophied septum could be observed in the longitudinal view of the histology (Figure 3B right panel), while the healthy septum showed well-aligned fiber pathways (Figure 3A, right panel), i.e., an appearance of smooth, coordinated muscle fiber alignment.

**FIGURE 4:**
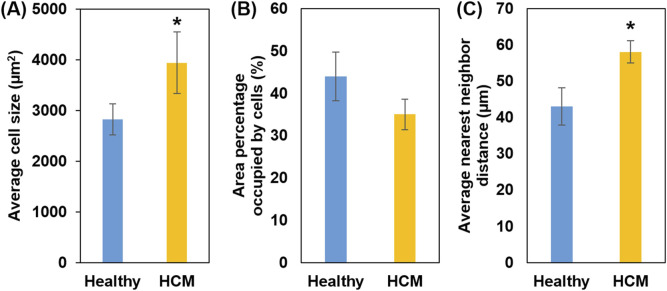
**(A)** Average cell size (cross-sectional area); HCM muscle fibers showed increased cell size. **(B)** Percentage of area occupied by heart muscle cells; smaller area percentage occupied by HCM muscle cells is consistent with the fact of overexpression of fibrotic ECM. **(C)** Nearest neighbor distance (NND): Higher NND in HCM reflects excess fibrotic ECM that further separates individual heart muscle cells, causing disorganization.

Quantified from the cross-sectional histological images, the average cell size of the hypertrophied cardiomyocytes (3,940 ± 610 μm^2^) was significantly larger than the cell size in the healthy septum (2,826 ± 304 μm^2^, *p < 0.05*, Figure 4A). We also calculated the percentage of area occupied by cardiomyocytes from the cross-sectional histological images and found that cardiomyocytes in the hypertrophied septum occupied less amount of area when compared to the healthy septum (healthy: 43.6% ± 5.8% vs. HCM: 34.8% ± 3.6%, Figure 4B). This observation is consistent with another key compositional abnormality in the hypertrophied septum, in which collagen was overexpressed in both endomysial and perimysial interstices. The overexpressed fibrotic ECM takes extra space and thus lowers the area percentage occupied by cardiomyocytes in the cross-sectional histology.

We also quantified the average nearest neighbor distance (NND) of cardiomyocytes in the cross-sectional histology (Figure 4C). NND reflects the spatial arrangement cells in a tissue, with a smaller NND indicating a denser cell arrangement, while a larger NND indicating a cell arrangement that is more dispersed and irregular. The analysis showed that the hypertrophied septum had significantly larger NND when compared with the healthy septum (healthy: 39.5 ± 5.1 µm vs. HCM 57.6 ± 3.1 µm, *p < 0.05*).

**FIGURE 5:**
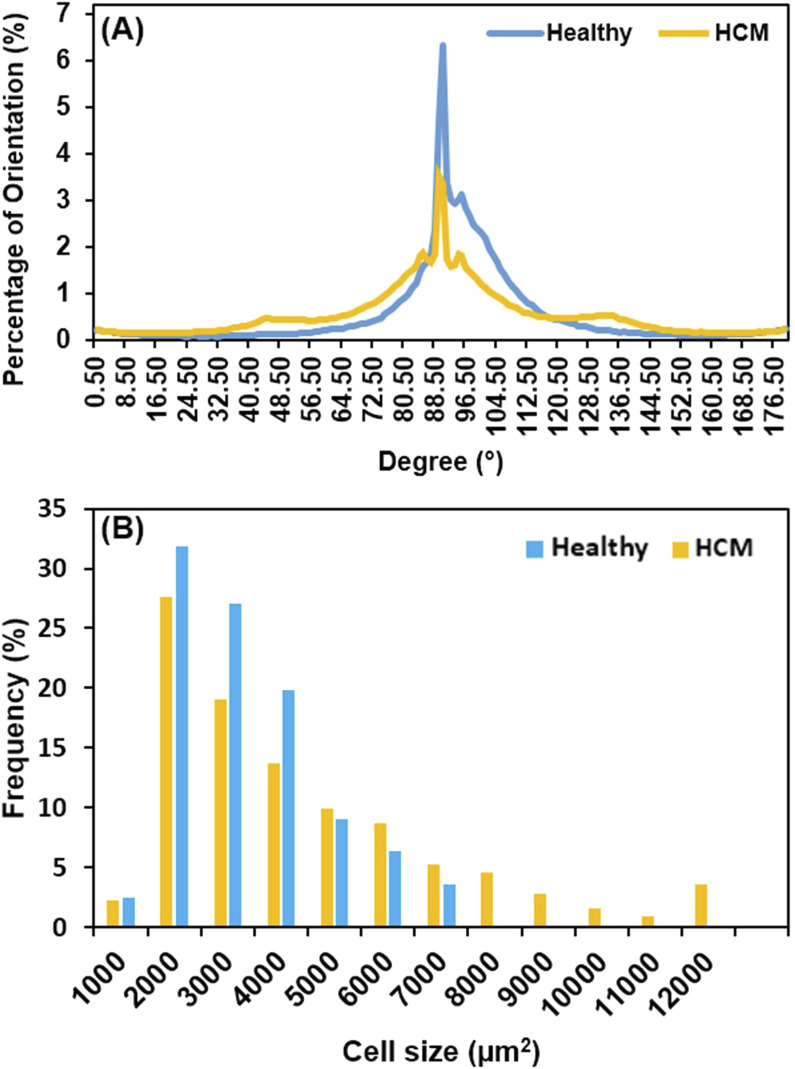
**(A)** Heart muscle fiber alignment quantified from the longitudinal view; the alignment of muscle fibers in HCM tissue was significantly more dispersed when compared with the healthy tissue. The loss of heart muscle alignment is one of the microstructural abnormalities of HCM. **(B)** Cell size distribution in frequency; HCM has a broader distribution, extending up to 12,000 μm^2^, reflecting a portion of normal-sized cells and pathologically enlarged cells due to hypertrophic growth.

Heart muscle fiber alignment quantified from the longitudinal histological images showed that the muscle fibers in hypertrophied septum were more dispersed and had a wider angular distribution of fiber orientation (Figure 5A). The spread of fiber angular distribution curves was consistent with the histological observation (Figure 3B), which showed that most healthy septum muscle fibers had an evenly distributed, coordinated alignment, while the hypertrophied septum muscle fibers demonstrated a less-organized, disarrayed morphology, another key microstructural abnormality in hypertrophic cardiomyopathy.

We further compared the cell size distribution in frequency between healthy septum and hypertrophied septum (Figure 5B). In healthy septum, most of cells (90.1%) had a size ranging from 1,000 μm^2^ to 5,000 μm^2^, with a small portion (9.9%) reaching up to 7,000 μm^2^. In the hypertrophied septum, the cells exhibited a broader size distribution, with 27.4% of the cells having an extremely enlarged size reaching up to 12,000 μm^2^. Additionally, we noticed was that there were 72.6% of cardiomyocytes with a size ranging from 1,000 μm^2^ to 5,000 μm^2^, indicating the cell size distribution curve shifted from left (smaller size) towards the right (larger size). In other words, we noted an overall trend of cellular enlargement, with a portion of the cells showing extreme dimensional increase. This observation, in which not every single cell experienced extreme enlargement, was consistent with previous genetic and cell biological observations (Ashrafian et al., 2007). Ashrafian et al. reported that sarcomere gene mutations experienced phenotypic expression in heterogenous manner, with some cardiomyocytes undergoing compensatory hypertrophy, while other cardiomyocytes exhibited no hypertrophic morphology and a portion of cardiomyocytes possibly underwent atrophy due to metabolic stress (Ashrafian et al., 2007).

### 3.5 2D section and 3D reconstruction of light-sheet microscopy images

**FIGURE 6:**
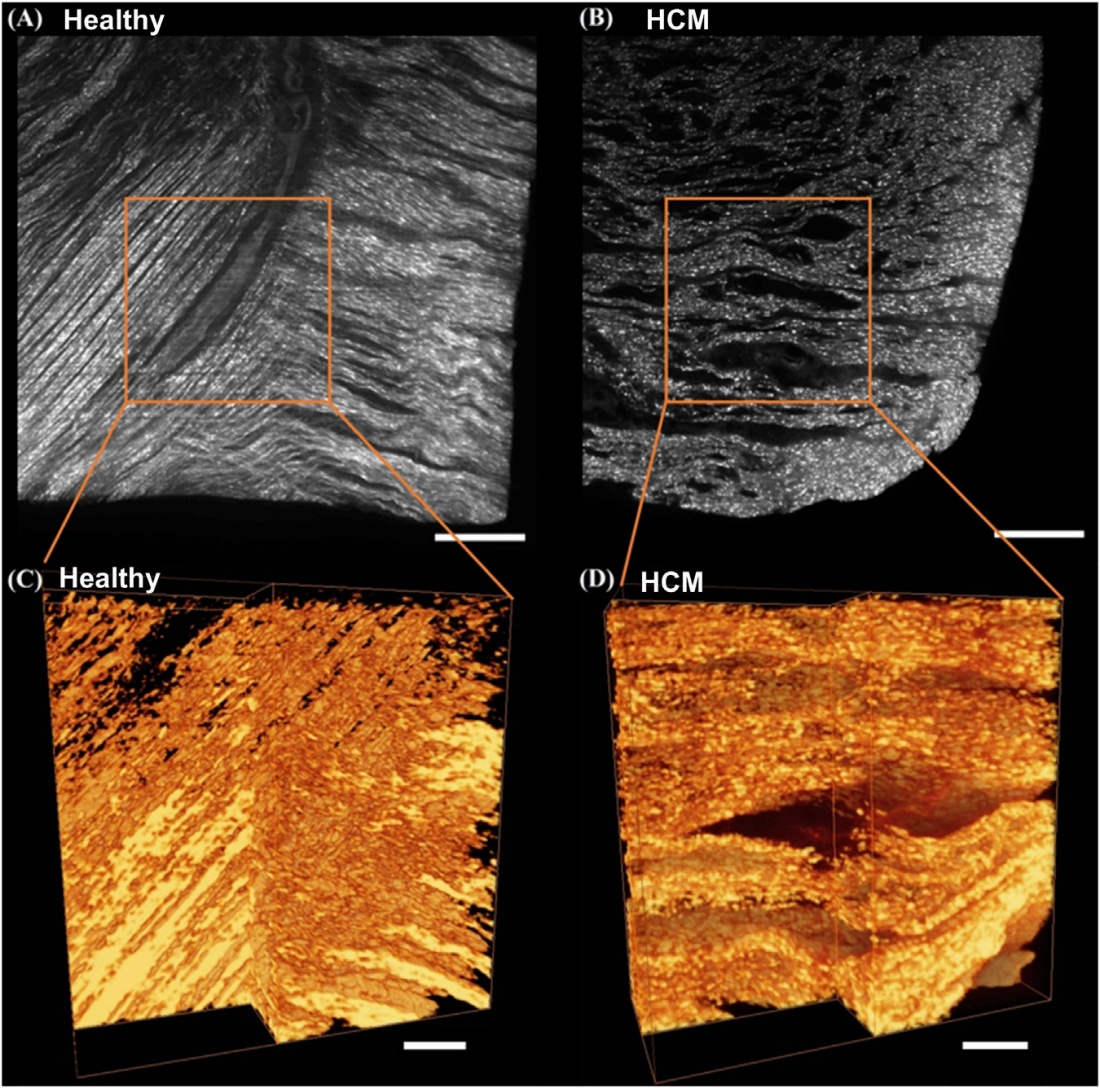
Light-sheet imaging of healthy human septum and hypertrophied septum. 2D section images of the healthy human septum **(A)** and hypertrophied septum **(B)**, scale bar: 500 μm; 3D reconstructed images of the healthy human septum **(C)** and hypertrophied septum **(D)**, scale bar: 200 µm.

As shown by light-sheet microscopy images (Figure 6), both in 2D section and 3D reconstruction, the healthy septum showed normal morphology (Figure 6A) with highly-aligned heart muscle fibers and evenly distributed small interfibrillar space, while the human hypertrophic septum showed disarrayed muscle fiber configurations (Figure 6B), with wider interfibrillar space that was unevenly distributed. The comparison of 3D reconstructed images further demonstrated that the disruption of the overall cell-collagen alignment and spatial distribution occurred in a 3D manner (Figures 6C,D).

## 4 Discussion

The biomechanical and microstructural findings in our study provide important insights into the pathophysiology of hypertrophic cardiomyopathy. We found that healthy human septum demonstrated viscoelastic behavior with typical characteristics of muscular tissues due to the interaction between cells, solid structural ECM microstructures, and hydrated ground substance (Humphrey et al., 1990; Novak et al., 1994; Pinto and Fung, 1973; Voorhees and Han, 2015). Under tensile load, the stress-strain curves of the healthy septum showed a nonlinear, concave upward trend (J-shaped curve), which was similar to the passive tensile behavior of heart muscles (Ahmad et al., 2018; Brazile et al., 2021; Wang et al., 2013; Zhang et al., 2010; Voorhees and Han, 2015; Holmes et al., 2005). The J-shaped curve represented an optimal soft tissue passive biomechanical behavior, in which a relatively shallow toe region, a gradually rising transitional region, and a sharp rising linear region provide flexible deformation at lower stress level, the needed stiffness for deformation locked-up at higher stress level, and failure-preventive mechanical strength. One can envision that, as the strain increased, the healthy septum gradually recruited a collagen network that was more organized at the ultrastructural level and well-integrated with heart muscle cells, and hence was able to achieve optimal nonlinear concave upward stress-strain curve shape, in which the fully recruited collagen fiber network in the linear region provided a locking-up mechanism to protect the muscle fibers from tearing and debonding.

In contrast to the healthy septum, the hypertrophied septum exhibited a less nonlinear stress-strain relation, a higher initial modulus in the lower strain region, decreased stiffness in the higher strain region, and greatly reduced mechanical strength (failure stress). The poorly organized, pathological collagen network in the hypertrophied septum seemed to lose the protective mechanisms as described above. However, the disorganized, overexpressed fibrotic collagen network in the hypertrophied septum did increase the initial tensile modulus, which unfortunately reduced flexible deformation in the physiological loading range and was an unfavorable tissue behavior.

The elevated initial modulus in HCM tissues aligns with prior reports of fibrosis-driven tissue stiffening due to collagen overexpression, and this increase of initial stiffness likely restricts early deformation (Marian and Braunwald, 2017). Moreover, the large reduction in maximum tensile modulus and failure stress suggests that this fibrotic overexpression/remodeling fails to confer structural resilience and mechanical strength. Instead, the disorganized collagen network and cardiomyocyte disarray might not yield high quality cell-collagen interweaving and might create stress concentration points, predisposing the tissue to premature failure (Ho et al., 2010). This conflicting duality, i.e., increased initial stiffness coupled with mechanical fragility at a higher loading range, might correlate with clinical observations of diastolic dysfunction and susceptibility to myocardial damage in HCM patients despite ventricular wall thickening (Ho et al., 2010; Maron et al., 2003; Wu et al., 2019).

Stress relaxation under tensile mode revealed that the healthy septum exhibited a stress decay trend similar to most soft tissues (Purslow et al., 1998; Liu et al., 2022), and the hypertrophied septum exhibited a much greater stress decay compared to healthy septum. The abundance of overexpressed fibrotic collagen matrix in the hypertrophied septum may be one of the major mechanism since interfibrillar sliding of collagen fibrils was observed to allow for greater stress decay in soft tissues (Szczesny and Elliott, 2014). Further quantification showed that the healthy septum exhibited faster short-term stress relaxation than the hypertrophied septum, suggesting efficient dissipation of stress immediately after strain application. We also noticed that the hypertrophied septum displayed a higher long-term relaxation rate, reflecting delayed stabilization compared to the healthy septum, which had a smaller long term relaxation rate, indicating healthy septum reaches equilibrium/stabilization earlier. A possible explanation is that the well-organized cell-collagen microstructures in the healthy septum enable efficient stress dissipation during the short-term relaxation and a quicker long-term response to reach tissue equilibrium. In contrast, the abnormal disorganized cell-collagen microstructures in the hypertrophied septum make short-term stress release more challenging and takes more time to reach stability and equilibrium, i.e., HCM’s unstable architecture prolongs stress relaxation. Both healthy septum and hypertrophied septum showed a small amount of creep over time, and the final creep of the hypertrophied septum was slightly higher than the healthy septum. More samples are needed to verify this trend in creep behavior. If the trend is verified with a larger sample size, the higher amount of creep in the hypertrophied septum can most likely be attributed to more abundant and more disarrayed fibrotic collagen fibers that are prone to creep.

Stress relaxation and creep behavior arise from the time-dependent microstructural rearrangement/adjustment of tissue ECMs and cells under sustained deformation and prolonged loading, respectively. Under physiologic condition, the normal stress relaxation behavior allows timely mitigation of a portion of the stress built up in tissue due to mechanical deformation; the normal creep behavior enables a small degree of compliance under prolonged loading condition. The viscoelastic abnormalities observed in the hypertrophied septum, however, underscore its microstructural dysfunction. The greater stress decay in hypertrophic septum implies impaired stress dissipation equilibrium status, potentially due to disrupted interactions between collagen and cardiomyocytes (Kraft et al., 2013). The biphasic relaxation behavior (slower short-term but faster long-term decay) in the hypertrophic septum may reflect altered solid-fluid interaction due to the deteriorated cell-collagen network, where fibrotic regions hinder rapid stress redistribution but allow prolonged stress relaxation (Fomovsky et al., 2010). While creep differences were not statistically significant, the trend toward greater creep deformation in hypertrophic septum aligns with the fact of collagen overexpression in pathology and warrants further investigation, as the creep behavior could contribute to progressive ventricular remodeling (Teekakirikul et al., 2010). The anisotropic stiffening observed in biaxial testing highlights the directional dependency of HCM remodeling. Longitudinal stiffening might impair ventricular filling by restricting chamber expansion, and disrupt wall stress distribution during the diastolic phase, underscoring the need for multiaxial mechanical assessments in HCM models (Sommer et al., 2015).

Our microstructural quantifications revealed the pathophysiological tissue configuration underlying the deteriorated biomechanical behavior of the hypertrophied septum. Heterogeneous cardiomyocyte enlargement and disarray, combined with disorganized collagen overexpression, create a mechanically inefficient tissue architecture (Ashrafian et al., 2007; Sommer et al., 2015; Fujiwara et al., 1983; Unverferth et al., 1987). These features mirror genetic studies that showed sarcomere mutations drive heterogeneous cardiomyocyte responses, with compensatory hypertrophy and atrophy coexisting in a metabolically stressed microenvironment (Ashrafian et al., 2007; Ho et al., 2010; Sommer et al., 2015; Bi et al., 2021). Consequently, the heterogeneous cell size distribution (with a subset of extremely hypertrophied cells) and disorganized overexpressed collagen network exacerbate regional stress imbalances, while increased nearest neighbor distance (NND) reflects ECM expansion and loss of cellular cohesion (Ho et al., 2010; Bi et al., 2021).

Due to the shape and size of myectomy tissue, it was challenging to prepare circumferential tissue strips for uniaxial mechanical tests. We thus did not perform these studies to attain tissue viscoelastic data along the circumferential direction of the septum. Future study to resolve this limitation will be pursued if the circumferential septum strips can be obtained via different protocols such as from deceased HCM hearts donated for research. Another limitation of our study was that the sample numbers for the tests were not enough for age-related analyses. Future studies are needed to investigate possible age-dependence of HCM tissue viscoelastic properties. Our study does fill a knowledge gap in HCM biomechanics by demonstrating how collagen overexpression, cellular hypertrophy, microstructural disorganization, and viscoelastic abnormalities collectively drive biomechanical deteriorations and functional vulnerabilities observed in hypertrophic cardiomyopathy. The obtained biomechanical and microstructural data can help derive human HCM-specific parameters and significantly improve the fidelity and clinical relevance of computational modeling and simulations related to HCM hearts. Moreover, our observations also imply that future therapies should target both fibrosis/ECM remodeling and cardiomyocyte mutations, in order to fully restore cardiac tissue biomechanical homeostasis.

## 5 Conclusion

Our study describes the deteriorated biomechanical properties in the human hypertrophied septum when compared with the healthy septum, as well as their underlying structural associations. The major discoveries are summarized as follows.

(1) The hypertrophied human septum was stiffer at the initial phase of tissue loading, but less nonlinear, less stiff in the linear region, and much weaker in mechanical strength when compared to healthy human septum. This conflicting duality, i.e., increased initial stiffness coupled with mechanical fragility at a higher loading range, might correlate with the clinical observations of diastolic dysfunction and susceptibility to myocardial damage in HCM patients despite ventricular wall thickening.
(2) The hypertrophied human septum exhibited significantly larger stress relaxation and slightly larger creep compared to the healthy human septum. Moreover, the abnormal, disorganized cell-collagen microstructures in the hypertrophied septum make short-term stress release more difficult and require longer relaxation time to reach equilibrium.
(3) Biaxial testing at lower stress levels revealed nonlinear anisotropic stress-strain behavior in both the healthy and hypertrophied septum, with the hypertrophied septum being stiffer in the longitudinal direction.
(4) Microstructural analysis via histology and light sheet microscopy imaging demonstrated the heterogeneous enlargement and disarray of cardiomyocytes, combined with disorganized collagen overexpression, led to a mechanically inefficient tissue architecture in the hypertrophied septum.

In short, the altered stress relaxation, creep, biaxial, and uniaxial tensile mechanical properties all reflect the fact that the hypertrophied septum has more heterogeneously enlarged, disarrayed cardiomyocytes and overexpressed, disorganized fibrotic collagen when compared with the healthy septum. The poorly organized, pathological composition of the hypertrophied results in inferior viscoelastic behavior compared to normal tissue. Our viscoelastic mechanical data and microstructural characterizations build a strong foundation to understand the altered tissue behavior of the hypertrophied septum, the degree of deviation from the normal septum, and the underlying structural mechanisms.

## Data Availability

The original contributions presented in the study are included in the article/supplementary material, further inquiries can be directed to the corresponding author.
